# Analysis of chemotherapy effect on the second primary malignancy for head and neck cancer patients by a nomogram based on SEER database

**DOI:** 10.1002/cam4.3442

**Published:** 2020-09-15

**Authors:** Xinrong Li, Kaibo Guo, Yuqian Feng, Yong Guo

**Affiliations:** ^1^ The First Clinical Medical College Zhejiang Chinese Medical University Hangzhou Zhejiang P. R. China; ^2^ Department of Medical Oncology The First Affiliated Hospital of Zhejiang Chinese Medical University Hangzhou Zhejiang P. R. China

**Keywords:** chemotherapy, head and neck cancer, nomogram, risk model

## Abstract

Second primary malignancy (SPM) ranks the second leading cause of death in patients with head and neck cancer (HNC), while studies exploring the risk factors for SPM are limited. To clarify this, we investigated the relationship between the chemotherapy and SPM using the Surveillance, Epidemiology, and End Results (SEER) database. 11 345 patients initially diagnosed with HNC between 1998 and 2016 were selected from the SEER database. First, these patients were divided into two groups according to chemotherapy or not. With Fine and Gray model, the subdistribution hazard ratio (sHR) of chemotherapy was calculated based on Propensity Score Matching (PSM). Second, the 11 345 cases were randomized into a training set and a validation set. Based on the training set, the different cumulative incidence of SPMs between the patients with and without chemotherapy was estimated respectively in the high‐ and low‐risk group according to the scores derived from a nomogram. Chemotherapy was negatively correlated to the SPMs (sHR: 0.847, 95% CI: 0.733‐0.977, *P* = .023) by conducting competing risk analysis. With chemotherapy, forest plots showed subgroups of squamous cell carcinoma (SCC, sHR: 0.815, 95% CI: 0.7‐0.948, *P* = .008), 50‐64 years old (sHR:0.794, 95% CI: 0.655‐0.962, *P* = .019), male (sHR:0.828, 95% CI: 0.703‐0.974, *P* = .023), and well/moderate histological grade (sHR:0.828, 95% CI: 0.688‐0.996, *P* = .045) were negatively correlated to SPMs; the nomogram showed the high‐risk population characterized as SCC, elder age, male, and well/moderate histological grade also tended to have lower incidence of SPMs (sHR: 0.805, 95% CI: 0.669‐0.969, *P* = .022). Despite HNC patients with characteristics of SCC, increased age, male, and well/moderate histological grade had higher risk of a SPM, they were also more likely to be benefitted from chemotherapy to avoid it.

## Graphical abstract

Second primary malignancy (SPM) ranks the second leading cause of death in patients with head and neck cancer. A conclusion was made that chemotherapy was negatively correlated with the SPM based on the comprehensive analysis of SEER database.

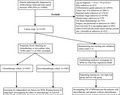

## 1 INTRODUCTION

Head and neck squamous cell carcinoma (HNSCC) is the sixth most prevalent cancer and one of the most aggressive malignancies with a high mortality rate worldwide.^1^ Owning to the high degree of biologic heterogeneity of head and neck carcinoma (HNC), it is a major challenge to implement an appropriate clinical management just according to the anatomical regions. Conventionally, surgery, radiation, and chemotherapy are common combinations used at the advanced stage of HNC, while surgery or radiation is usually applied at the limited or early‐stage disease.^2^ In the recent two decades, great efforts such as new cytotoxic agents, anti‐EGFR monoclonal antibody,^3^ and immune checkpoint inhibitors^4^ have been developed in order to prolong the survival of HNSCC, but the prognosis is till poor.^5^

It should be noted that second primary malignancy (SPM) is the second leading cause of death in patients with HNSCC,^6^ nearly 1/4‐1/3 of deaths in these patients are attributable to SPM,^7^ which highlights the importance of successful management of SPM in patients with HNSCC besides focusing on the aggressive and multiple treatment for the initial malignancies. Numerous studies have been performed to discuss the relationship between the radiotherapy and the SPMs,^8^, ^9^ and most views hold that radiation therapy is a contributing factor to carcinogenesis. However, few studies were conducted to investigated the correlations between chemotherapy and new tumors occurrence in HNC.

In the present study, based on a postmatch cohort created by Propensity Score Matching (PSM),^10^ we discussed the relations between chemotherapy and SPMs, and identified its effect on the SPMs of patients with some characteristics. With consideration of selective bias^11^ and ensuring the integrity of real‐world data from SEER database, a nomogram was developed from the prematch cohort to divide the population into high/low‐risk groups by some predictive factors, then the effect of chemotherapy on the SPMs in the above groups was estimated. Moreover, the correlations between chemotherapy and overall survival were further studied.

## 2 MATERIALS AND METHODS

### 2.1 Study design

Retrospectively reviewing data in the Surveillance, Epidemiology, and End Results (SEER) database from 1998 to 2016, cases histologically diagnosed with initial HNC according to the International Classification of Diseases in Oncology, third edition [ICD‐O‐3] were selected. All patients were characterized by gender, race, age at diagnosis, detailed anatomical site of HNC, histology, grade, tumor size, lymph nodes status, cause of death, disease stage, marital status at diagnosis and chemotherapy recode. A SPM is defined as a second malignancy that presents either a synchronous SPM or a metachronous SPM according to the intervals within or greater than 6 months after the primary tumor.^12^ Then, the information of a metachronous SPM for each case was also selected if available. Because of the heterogeneity of HNC, ‘SEER Summary stage 2000’ variable was selected instead of the AJCC staging system. This classification standard classified cancer cases as localized, regional, and distant. And for the numerous changes in cancer staging over the previous three decades, “EOD 10 ‐ size (1988‐2003),” “CS tumor size (2004‐2015),” or “Tumor Size Summary (2016+)” variable was used respectively to measure the accurate size of the initial HNC. Patients with missing information were excluded from the analysis. Survival time was defined as months from diagnosis to death or last follow‐up if alive.

### 2.2 Statistical analysis

We used R, version 3.6.1 (http://www.r‐project.org/) software for statistical analysis. Descriptive statistics for each variable were reported. Categorical variables were compared using the χ^2^ test or Fisher exact test. All statistical tests were two sided, and a *P* value <.05 was considered statistically significant.

The patients with performed surgery and beam radiotherapy were divided into two groups, one included the crowd who received chemotherapy and the another contained those who did not. Using PSM, patients with chemotherapy were matched to those without chemotherapy at a ratio of 1:1 to balance baseline characteristics (race, age at diagnosis, gender, histologic type, tumor size, lymph nodes, tumor stage, and marital status) by the caliper value of 0.001. After matching, the balance of variates between two groups was evaluated by the χ^2^ test and love‐plot, P value of >0.05 in χ^2^ test or plots within two dashed vertical lines in love‐plot was considered as balance.^13^ In the postmatch cohort, univariate and multivariate competing risk regression model was used to estimate the subdistribution hazard ratio (sHR) of variates such as age, gender, race, marital status, site of tumor, histological type, differentiation grade, tumor size, lymph node status, and summary stage. Forest plots were created to better present the effect of chemotherapy on cumulative incidence of SPMs in different subgroups. Furthermore, Kaplan‐Meier estimation was performed to indicate whether the chemotherapy could affect the survival outcomes or not.

To further investigate the effect of chemotherapy on SPMs without selective bias, 11 345 patients in the prematch cohort were randomized into a training cohort and a validation cohort at a ratio of 7:3. Significant statistical variables identified by multivariate competing risk regression analysis and clinicopathological variables^14^ assessed from previously published articles were used to establish a nomogram. To measure the discrimination and calibration of the nomogram both in the training and validation cohorts, a concordance index (C‐index)^15^ was calculated and calibration curves were drawn with a bootstrap approach involving 500 resamples. The 3‐, 5‐, and 10‐year calibrations were performed to compare the predicted incidence of SPMs to the observed one. If the model calibration is ideal, dots on the calibration plot should be close to a 45° diagonal line.^16^ Total score of each patient was estimated by nomogram, which was corresponded to the risk of SPMs, then all patients could be divided into high‐ and low‐risk groups by the median of the risk scores. Then, the sHR of chemotherapy (vs nonchemotherapy) was calculated and survival analysis after PSM (ratio: 1:1; caliper value: 0.001) was conducted for the above groups, respectively.

## 3 RESULTS

### 3.1 Baseline characteristics

**FIGURE 1:**
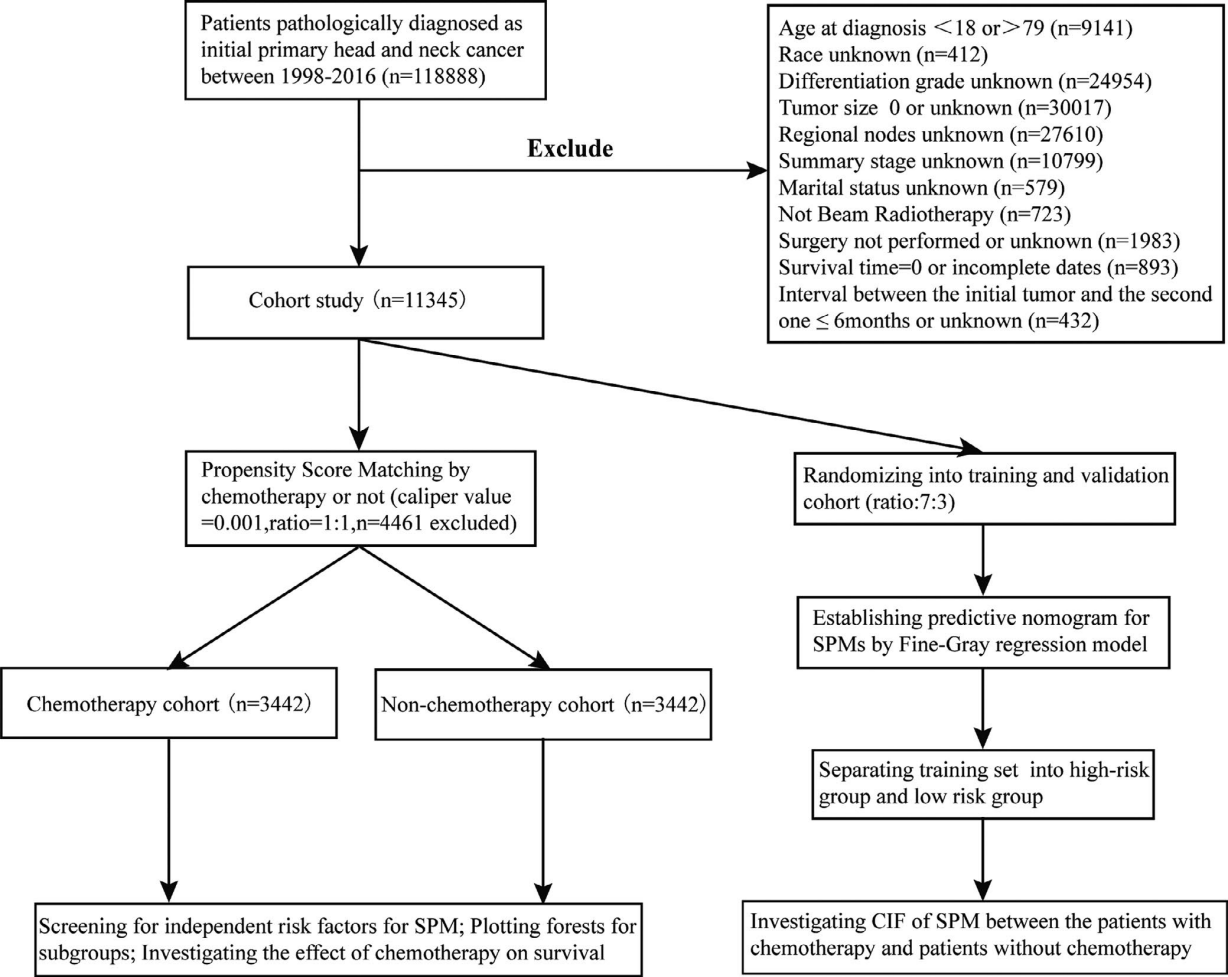
The data selection steps of the present study

As Figure 1 showed, totaling of 118 888 patients were initially identified from the SEER database. Subsequently, the patients with age of <18 or >79 (n = 9141), absent beam radiotherapy (n = 723), unperformed surgery or unknown (n = 1983), short (≤6 months) or unknown interval between the initial tumor and the second one (n = 432), missing survival time (n = 893) or other incomplete information including tumor size(n = 30 017), nodes quantities (n = 27 610), race (n = 412), differentiation grade (n = 24 954), marital status (n = 579), and stage (n = 10 799) were excluded. As a result, 11 345 eligible patients with initial primary HNC were selected.

With performing PSM, totaling of 4461 patients were excluded at match conditions of ratio (1:1) and caliper value (0.001), and a total of 6884 cases, including 3442 cases with chemotherapy and 3442 cases without chemotherapy, were finally selected for the further analysis. The characteristics of the patients before and after match were presented in Table 1. As it showed, only marriage status (*P* = .629) and race (*P* = .662) had similar distributions between the chemotherapy group and the nonchemotherapy group before match. And after PSM assessed by love‐plot (Figure S1A), the distribution of all characteristics became similar, indicating a good comparability between the two groups. For example, in the postmatch cohort, among 3442 cases with chemotherapy, the majorities of clinical characteristics were male (2612, 75.89%), white race (2855, 82.95%), 50‐64 years old (1896, 55.08%), married (1924, 55.90%), site of tongue (1618, 47.01%), squamous cell carcinoma (SCC, 2965, 86.14%), well or moderate differentiation (1955, 56.80%), smaller tumor size (≤31 mm, 1796, 52.18%), positive node status (2715, 78.88%), and regional stage (2260, 65.66%). The similar distribution was also observed in the nonchemotherapy group of postmatch cohort.

**TABLE 1:** Clinical characteristics before and after matching based on the propensity score

Variables	Before matching		*P* value^a^	After matching		*P* value^a^
	Chemotherapy n = 4455 (%)	Nonchemotherapy n = 6890 (%)		Chemotherapy n = 3442 (%)	Nonchemotherapy n = 3442 (%)	
Age						
18‐49 year	961 (21.57%)	1479 (21.47%)	＜.001	627 (18.22%)	651 (18.91%)	.578
50‐64 year	2458 (55.17%)	3273 (47.50%)		1896 (55.08%)	1854 (53.86%)	
65‐79 year	1036 (23.25%)	2138 (31.03%)		919 (26.70%)	937 (27.22%)	
Gender						
Male	3376 (75.78%)	4794 (69.58%)	＜.001	2612 (75.89%)	2630 (76.41%)	.611
Female	1079 (24.22%)	2096 (30.42%)		830 (24.11%)	812 (23.59%)	
Race						
White	3593 (80.65%)	5509 (79.96%)	.662	2855 (82.95%)	2867 (83.29%)	.592
Black	546 (12.26%)	876 (12.71%)		390 (11.33%)	397 (11.53%)	
Others^b^	316 (7.09%)	505 (7.33%)		197 (5.72%)	178 (5.17%)	
Marriage						
Married	2518 (56.52%)	3926 (56.98%)	.629	1924 (55.90%)	1950 (56.65%)	.528
Unmarried	1937 (43.48%)	2964 (43.02%)		1518 (44.10%)	1492 (43.35%)	
Site						
Lip and mouth	531 (11.92%)	910 (13.21%)	＜.001	457 (13.28%)	412 (11.97%)	.145
Salivary glands	679 (15.24%)	2054 (29.81%)		555 (16.12%)	589 (17.11%)	
Larynx	1001 (22.47%)	1543 (22.39%)		812 (23.59%)	863 (25.07%)	
Tongue	2244 (50.37%)	2383 (34.59%)		1618 (47.01%)	1578 (45.85%)	
Histology						
Squamous	3893 (87.38%)	5054 (73.35%)	＜.001	2965 (86.14%)	2972 (86.35%)	.807
Others^c^	562 (12.62%)	1836 (26.65%)		477 (13.86%)	470 (13.65%)	
Grade ^d^						
Ⅰ+Ⅱ	2355 (52.86%)	4289 (62.25%)	＜.001	1955 (56.80%)	1993 (57.90%)	.354
Ⅲ+Ⅳ	2100 (47.14%)	2601 (37.75%)		1487 (43.20%)	1449 (42.10%)	
Size						
≤30 mm	2145 (48.15%)	3979 (57.75%)	＜.001	1796 (52.18%)	1765 (51.28%)	.455
≥31 mm	2310 (51.85%)	2911 (42.25%)		1646 (47.82%)	1677 (48.72%)	
Lymph node						
Negative	793 (17.80%)	3235 (46.95%)	＜.001	727 (21.12%)	735 (21.35%)	.814
Positive	3662 (82.20%)	3655 (53.05%)		2715 (78.88%)	2707 (78.65%)	
Stage						
Localized	238 (5.34%)	1701 (24.69%)	＜.001	229 (6.65%)	237 (6.89%)	.877
Regional	2726 (61.19%)	3612 (52.42%)		2260 (65.66%)	2242 (65.14%)	
Distant	1491 (33.47%)	1577 (22.89%)		953 (27.69%)	963 (27.98%)	

^a^ The *P* values of comparing chemotherapy and nonchemotherapy calculated with the use of a chi‐square test.

^b^ Other race (American Indian/AK Native, Asian/Pacific Islander).

^c^ Other histological types (larger/small cell carcinoma, adenocarcinoma, sarcoma, neuroendocrine carcinoma, et.al).

^d^ well differentiation; Ⅱ, moderate differentiation; Ⅲ, poor differentiation; Ⅳ, undifferentiation.

### 3.2 Analysis in postmatch cohort

**FIGURE 2:**
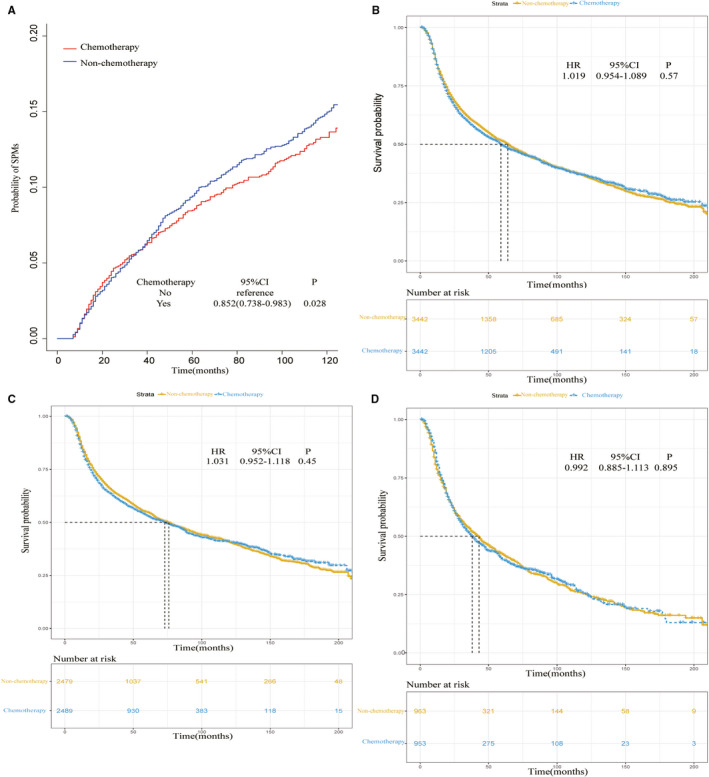
A, Cumulative incidence estimates of SPM for patients with or without chemotherapy in the postmatch cohort; B, Overall survival of the whole‐population with or without chemotherapy in the postmatch cohort. C, Survival comparison between chemotherapy and non‐chemotherapy for patients with non‐advanced disease. D, Survival comparison between chemotherapy and non‐chemotherapy for patients with advanced disease

SHR of chemotherapy for SPM was estimated by the univariate and multivariate analysis of Fine‐Gray regression model. The sHRs of chemotherapy were 0.852 (95% CI: 0.738‐0.983, *P* = .028) in the univariate analysis and 0.847 (95% CI: 0.733‐0.977, *P* = .023) in the multivariate analysis respectively (Table 2), showing its significant negative correlation with occurrence of SPMs (Figure 2A).

**TABLE 2:** SHR of chemotherapy for SPMs in Univariate and Multivariate competing risk models

Chemotherapy	Univariate analysis			Multivariate analysis		
	sHR^a^	95% CI^b^	*P* value	sHR^a^	95% CI^b^	*P* value
No	1(Ref)			1(Ref)		
Yes	0.852	0.738‐0.983	.028	0.847	0.733‐0.977	.023

^a^ sHR, subdistribution hazard ratio.

^b^ CI, confidence interval.

**FIGURE 3:**
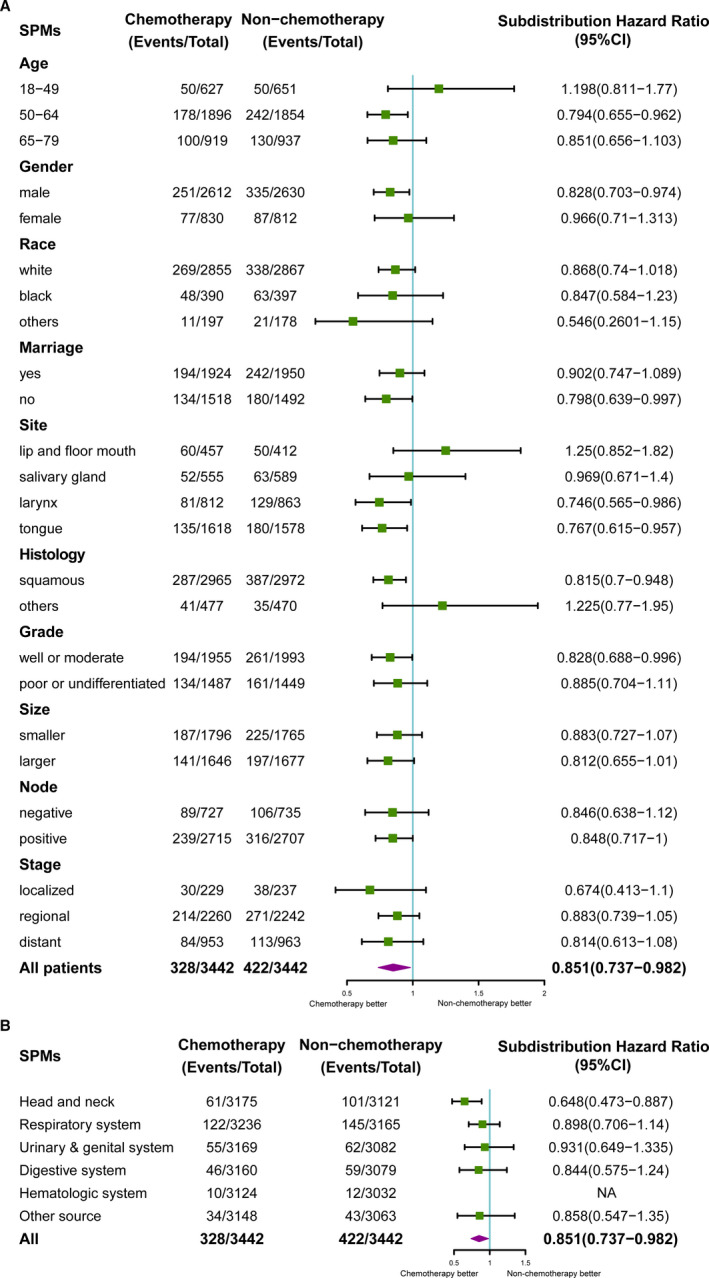
A, Multivariate competing risk analysis of SPM in HNC patients by forest plots. The X‐axis shows the sHR and 95% CI of chemotherapy in each subgroup, ticks follow the arrangement of 0.5, 1.0, 1.5 and 2.0；B, Multivariate competing risk analysis of SPM in HNC patients by forest plots. The X‐axis shows the sHR and 95% CI of chemotherapy in each subgroup, ticks follow the arrangement of 0.5, 1.0 and 1.5. (Value of ‘hematologic system’ was 'NA' because of limited samples in this subgroup)

Forest plots were used to display the effects of chemotherapy in different subgroups for SPMs. As Figure 3A showed, significant sHRs (chemotherapy vs nonchemotherapy) could be observed in some subgroups such as 50‐64 years old (sHR:0.794, 95% CI:0.655‐0.962, *P* = .019), male (sHR:0.828, 95% CI: 0.703‐0.974, *P* = .023), unmarried status (sHR: 0.798, 95% CI: 0.639‐0.997 *P* = .047), site of larynx (sHR: 0.746, 95% CI: 0.565‐0.986, *P* = .039), sit of tongue (sHR: 0.767, 95% CI: 0.615‐0.957, *P* = .019), SCC (sHR: 0.815, 95% CI: 0.7‐0.948, *P* = .008) and well/moderate grade (sHR:0.828, 95% CI:0.688‐0.996, *P* = .045), which demonstrated that the patients with characteristics mentioned above were more likely to be benefitted from the chemotherapy to reduce the incidence of SPMs. As for the origins of SPMs, the top 10 types of SPMs were originated from lung and bronchus, prostate, tongue, esophagus, gum and other in mouth, urinary bladder, breast, miscellaneous, stomach, melanoma of the skin, accounting for 72.93% of all SPMs in our study (Figure S2). Significant sHR of chemotherapy vs nonchemotherapy was only observed in the subgroup of “Head and neck region” (sHR: 0.648, 95% CI: 0.473‐0.887, *P* = .007, Figure 3B).

Furthermore, no significant difference of survival prognosis was observed between the patients with and without chemotherapy either in whole‐population cohort (HR: 1.019, 95% CI: 0.954‐1.089, *P* = .57, Figure 2B) or stage‐based subgroups (nonadvanced stage: HR: 1.031, 95% CI: 0.952‐1.118, *P* = .45, Figure 2C; advanced stage: HR: 0.992, 95% CI: 0.885‐1.113, *P* = .895, Figure 2D).

### 3.3 Analysis in prematch cohort

As Table 3 showed, based on the training set, the univariate analysis showed that age, race, tumor site, histological type, histological grade, tumor size, lymph node status, and summary stage were significantly correlated to SPMs (*P* < .05). Subsequently, the multivariate analysis indicated that factors such as site of tongue (vs site of Lip and mouth, sHR: 0.696, 95% CI: 0.571‐0.849, *P* < .001), tumor size of ≥31 mm (vs ≤30 mm, sHR: 0.848, 95% CI: 0.736‐0.977, *P* = .022), distant stage (vs localized stage, sHR: 0.775, 95% CI: 0.605‐0.992, *P* = .043) and node positive (vs negative, sHR: 0.825, 95% CI: 0.694‐0.981, *P* = .03) were accompanied by lower incidences of SPMs. While some other factors for increased age (50‐64y vs 18‐49y, sHR: 1.666, 95% CI: 1.364‐2.034, *P* < .001; 65‐79y vs 18‐49y, sHR: 2.133, 95% CI: 1.726‐2.636, *P* < .001), black race (vs white race, sHR: 1.369, 95% CI: 1.14‐1.644, *P* < .001), and SCC (vs nonsquamous, sHR: 1.483, 95% CI: 1.112‐1.979, *P* = .007) were positive correlated with SPMs.

**TABLE 3:** SHR of characteristics for SPMs in Univariate and Multivariate competing risk models

Characteristics	Univariate analysis			Multivariate analysis		
	sHR^a^	95% CI^b^	*P* value	sHR^a^	95% CI^b^	*P* value
Age						
18‐49 year	1(Ref)			1(Ref)		
50‐64 year	1.66	1.36‐2.02	＜.001	1.666	1.364‐2.034	＜.001
65‐79 year	2.09	1.7‐2.57	＜.001	2.133	1.726‐2.636	＜.001
Race						
White	1(Ref)			1(Ref)		
Black	1.299	1.087‐1.55	.004	1.369	1.14‐1.644	＜.001
Others^c^	0.875	0.663‐1.15	.34	0.989	0.749‐1.306	.94
Gender						
Male	1(Ref)					
Female	0.878	0.754‐1.02	.096			
Marriage						
Unmarried	1(Ref)					
Married	1.07	0.938‐1.23	.3			
Site						
Lip and mouth	1(Ref)			1(Ref)		
Salivary glands	0.641	0.519‐0.792	＜.001	0.896	0.666‐1.206	.17
Larynx	0.773	0.632‐0.946	.012	0.82	0.66‐1.019	.074
Tongue	0.625	0.516‐0.757	＜.001	0.696	0.571‐0.849	＜.001
Histology						
Others^d^	1(Ref)			1(Ref)		
Squamous	1.32	1.11‐1.58	.002	1.483	1.112‐1.979	.007
Grade^e^						
Ⅰ + Ⅱ	1(Ref)			1(Ref)		
Ⅲ + Ⅳ	0.835	0.729‐0.957	.01	0.887	0.768‐1.025	.1
Size						
≤30 mm	1(Ref)			1(Ref)		
≥31 mm	0.858	0.75‐0.981	.025	0.848	0.736‐0.977	.022
Lymph node						
Negative	1(Ref)			1(Ref)		
Positive	0.788	0.689‐0.901	＜.001	0.825	0.694‐0.981	.03
Stage						
Localized	1(Ref)			1(Ref)		
Regional	0.861	0.725‐1.023	.09	0.918	0.735‐1.147	.45
Distant	0.751	0.615‐0.919	.0053	0.775	0.605‐0.992	.043

^a^ sHR, subdistribution hazard ratio.

^b^ CI, confidence interval.

^c^ Other race (American Indian/AK Native, Asian/Pacific Islander).

^d^ Other histological types (larger/small cell carcinoma, adenocarcinoma, sarcoma, neuroendocrine carcinoma, et.al).

^e^ Ⅰ, well differentiation; Ⅱ, moderate differentiation; Ⅲ, poor differentiation; Ⅳ, undifferentiation.

**FIGURE 4:**
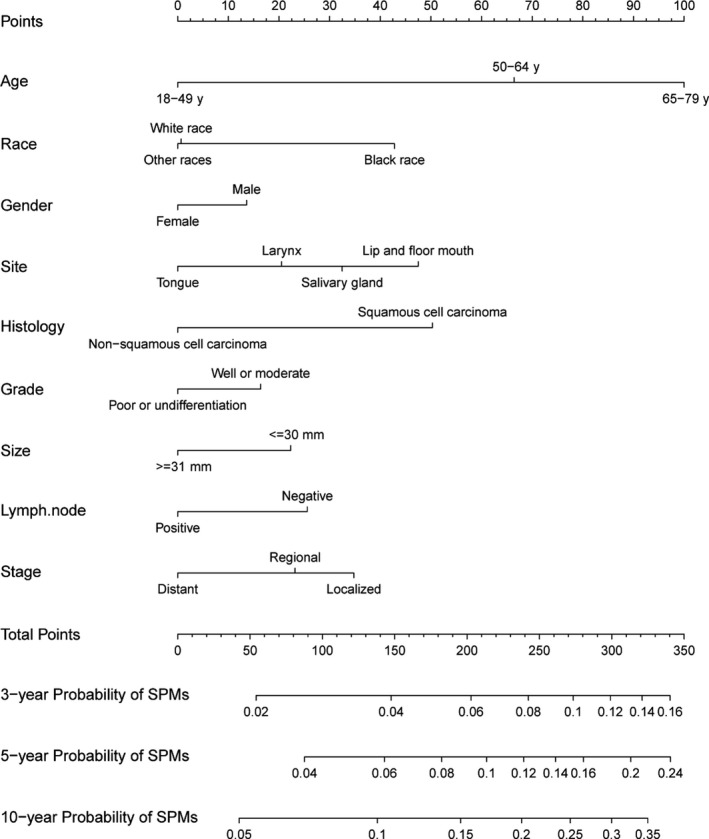
Nomogram predicting 3‐, 5‐, and 10‐year probabilities of SPMs for HNC patients based on training cohort

**FIGURE 5:**
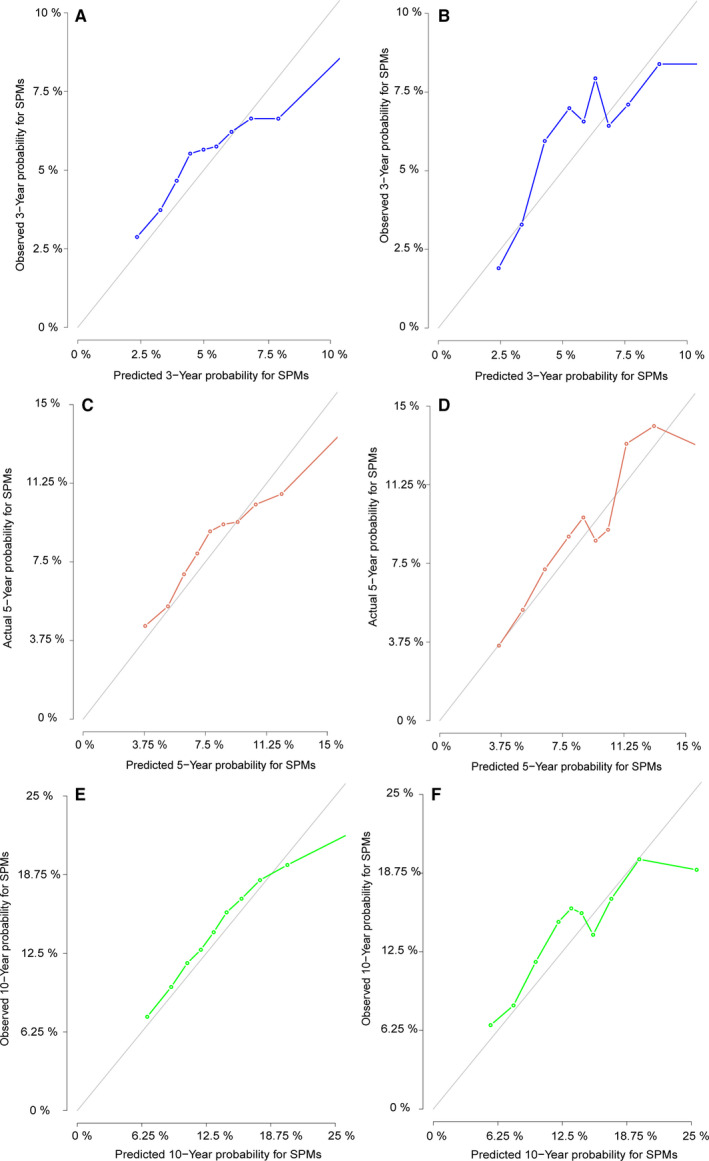
A, C and E, The calibration curves of nomogram for predicting 3‐, 5‐, and10‐year probabilities of SPM in the training set. B, D and F, The calibration curves of nomogram for predicting 3‐, 5‐, and 10‐year probabilities of SPM in the validation set. Nomogram‐predicted SPM is plotted on the x‐axis; actual SPM is plotted on the y‐axis. The imaginary line indicates a perfect calibration model in which the predicted probabilities are identical to the actual incidence

With the independent predictive factors selected from the multivariate competing risk regression model and two additional factors for “gender” and “stage” derived from the precious published article,^17^ a nomogram was established to display the 3‐year, 5‐year, and 10‐year probabilities of SPMs (Figure 4). The C‐indexes in the training and validation cohorts were 0.631 (95% CI: 0.611‐0.651) and 0.636 (95% CI: 0.607‐0.665) respectively, representing the moderate discrimination ability of the nomogram. The calibration curves of 3‐year, 5‐year, and 10‐year based on training and validation cohort were shown in Figure 5. It appeared that the calibration curves were all very close to the ideal curves, representing the good agreements between the nomogram‐predicted and the actual 3‐, 5‐, and 10‐year SPMs' incidence. That is to say, the nomogram has good predictive accuracy and reliability in predicting 3‐, 5‐, and 10‐year incidences of SPMs for patients initially diagnosed with HNC.

**FIGURE 6:**
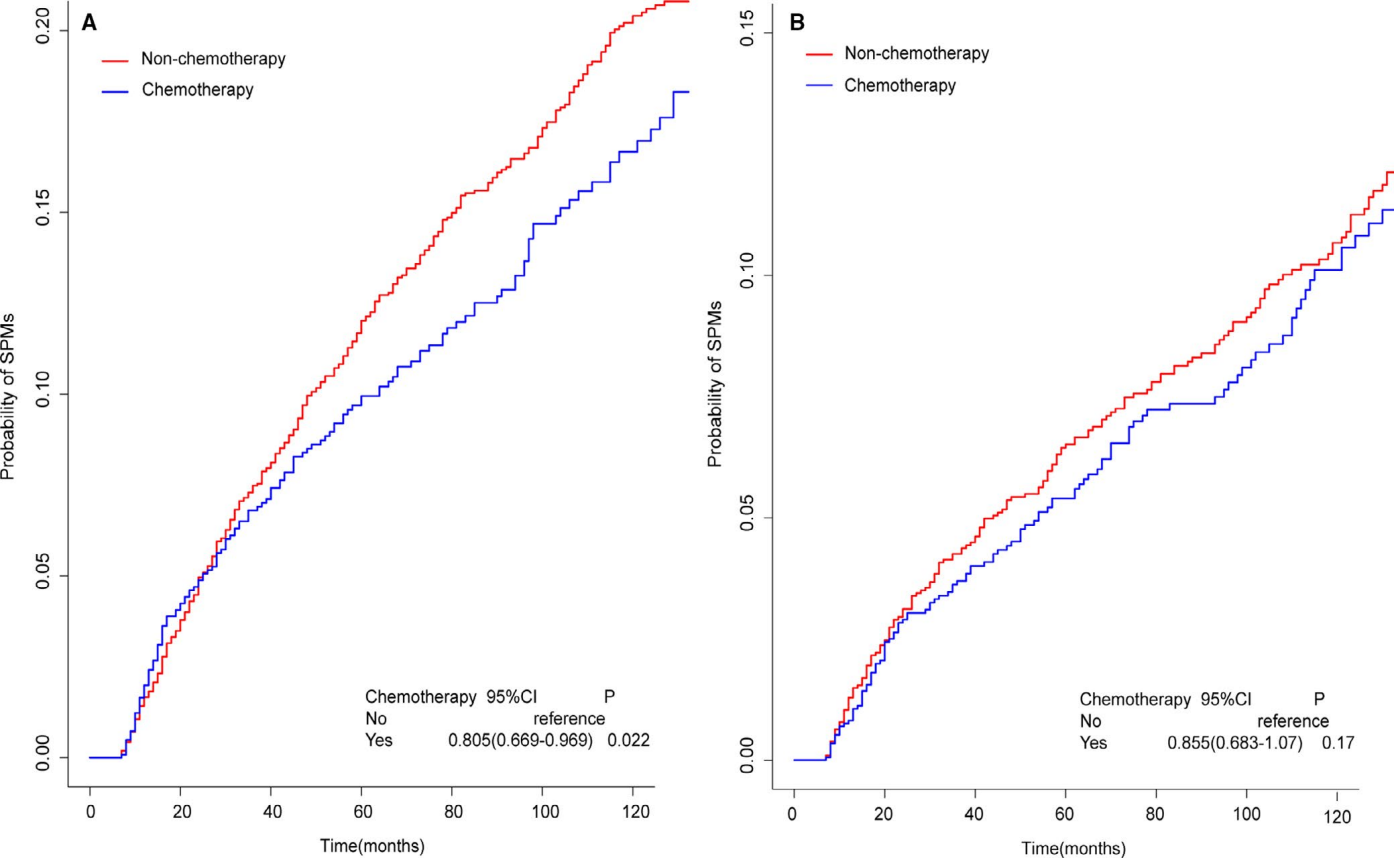
A, Cumulative incidence estimates of SPMs for patients with or without chemotherapy in high‐risk group；B, Cumulative incidence estimates of SPMs for patients with or without chemotherapy in lowrisk group

**FIGURE 7:**
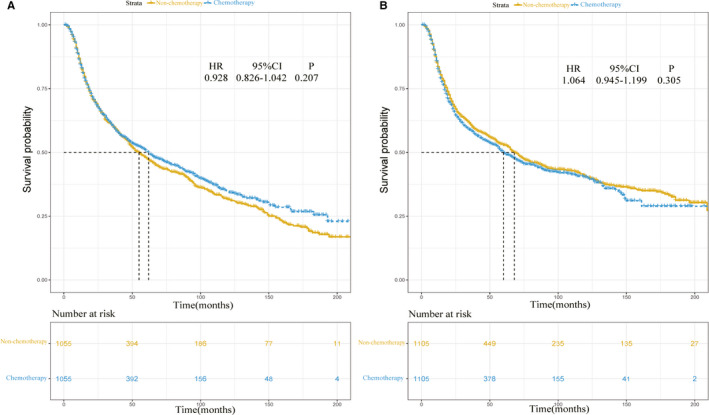
A, Overall survival of HNC patients with or without chemotherapy in the high‐risk group; B, Overall survival of HNC patients with or without chemotherapy in the low‐risk group

Using the nomogram, for different variables pointed to a specific score according to the top scale and then a total score by summing up all scores for each patient could be calculated. After that, the training cohort was divided into high‐ and low‐risk groups by the median value of 189 (low, ≤189 vs high, >189). The patients with chemotherapy in the high‐risk group were significant negatively correlated to the occurrence of SPMs (sHR: 0.805, 95% CI: 0.669‐0.969, *P* = .022, Figure 6A), while no significant difference was observed in the low‐risk group between the patients received chemotherapy and those did not (sHR: 0.855, 95% CI: 0.683‐1.07, *P* = .17, Figure 6B). Finally, after propensity‐matching assessed by love‐plot (Figure S1B and C), the survival analysis was performed in high‐ and low‐risk group by Kaplan‐Meier approach. As a result, no significant difference was observed either in the high‐risk group (HR: 0.928, 95% CI: 0.826‐1.042, *P* = .207, Figure 7A) or low‐risk group (HR: 1.064, 95% CI: 0.945‐1.199, *P* = .305, Figure 7B).

## 4 DISCUSSION

The incidence of SPMs after a diagnosis of HNC is about 3%‐7% per year,^18^ ranking the highest level among solid tumors.^19^ Several studies have discussed the epidemiology and risk factors for SPMs based on the demographic, diagnostic, and treatment factors of HNC survivors. The factors include smoking, alcohol consumption, human papillomavirus (HPV) infection (especially for oropharyngeal cancers), and Epstein‐Barr virus (EBV) infection.^20^ Previous treatment for HNC using external beam radiation therapy is associated with a decreased incidence of SPMs within the treatment fields,^21^ while exposure to radiation therapy may be associated with various cancers in nonirradiated areas, such as thyroid cancer or sarcoma.^22^ In the present study, we have showed some predictive factors for metachronous SPMs of patients with HNC, and as far as we know, this is the first time to comprehensively discuss the relationship between chemotherapy and occurrence of SPMs.

According to the study, some factors referring to histological type, gender, age, lymph node status, and tumor stage were found to be significantly correlated to SPMs. SCC was viewed as a risk factor to develop SPMs in our study. It is accordance with the fact that the incidence of SPMs in HNSCC is high, accounting for the 20‐year cumulative risk of 36%.^20^ The possible reasons for this phenomenon are considered as tobacco smoking, alcohol drinking, and HPV infection.^18^ As for gender, men were more likely to develop SPMs than women. Through review of 59,958 cases from the Thames Cancer Registry (TCR) database, Warnakulasuriya et.al pointed out an increased male‐to‐female ratio of SPMs in HNC patients with the reasons that tobacco smoking was more prevalence in males.^23^ Previous studies reported that the risk of SPMs was increased in young patients with HNC, and this risk decreased with increasing age,^20^ for the younger man had more time to develop a SPM. However, the conclusion is not consistent. Iwatsubo et al revealed the cumulative incidence of the SPMs(except esophageal cancer) in young patients was significantly lower than that in old patients (7.8% vs 12.2% at 5 years, and 13.9% vs 15.3% at 10 years; *P* = .017).^24^ Milano et al indicated younger age (*P* = .060) for the second irradiated HNC (HR: 0.75, 95% CI: 0.55‐1.01, *P* = .062) was a significantly favorable risk factor.^25^ Besides, higher risk of SPMs was observed among the elderly patients in our study. Although it is difficulty to clarify the causal relationship in a retrospective cohort with high proportion of the elder, a possible reason for this result is that the old seem to have less ability to repair somatic DNA damage, as a result, potential mutation is accumulated to promote the cancerization.^26^ As reported previously, black race was an independent risk factor to develop SPMs. Because black men have higher rates of socioeconomic barriers to receive timely, high quality medical care.^27^ Interestingly, comparing with other races, the black HNC patients were less prone to suffer from SPMs when they received chemotherapy previously, which needed to be confirmed by further large‐scale clinical observation. Usually, positive lymph node and distant stage are correlated to the poor diagnosis. While in our study, these two factors as well as chemotherapy were negatively related with SPMs. The probable reason is that these patients often receive combinations of different cytotoxic drugs for eliminating the cancerization lesions to a large extent. Field cancerization,^28^ usually resulting from long‐term smoking and drinking,^18^ is a popular theory of SPMs origin. Numerous agents such as vitamin,^29^ synthetic retinoids,^30^ cyclooxygenase‐2 inhibitors,^31^ epidermal growth factor receptor inhibition,^32^ and immune checkpoint inhibitor^33^ have been studied as potential chemo‐preventive agents. Unfortunately, none of these agents has been shown significant efficacy in large randomized clinical trials. In this retrospective study, we found the chemotherapy was negatively correlated to SPMs. To some extent, cytotoxic drugs could be viewed as chemo‐preventive agents. For instance, metronomic chemotherapy could exert positive effect on the stimulation of the antitumor immune response,^34^ and an activated immune system plays a pivotal role in cancer prevention, development, and defense.^35^

Based on a postmatch cohort, competing risk regression model represented that chemotherapy was negatively related to the SPM. The subgroup analysis was displayed by forest plots, indicating patients with some characteristics such as SCC, middle age (50‐64 years old), male, well or moderate histological grade, unmarried status, and site of tongue were more likely to be benefitted from chemotherapy for lower incidence of the SPMs primarily originated from head and neck regions (Figure 3A and B). Similarly, based on a training set derived from prematch cohort, a nomogram with good discrimination and calibration was established, showing the high‐risk group characterized as SCC, elder age, male, well or moderate histological grade, black race, smaller size, negative node and localized stage was less likely to develop a SPM with the help of chemotherapy. With the results from post‐ and prematch cohorts, it could be deduced chemotherapy played a positive role in preventing SPMs for the patients with some characteristics of SCC, increased age, male, and well/moderate histological grade.

Finally, we estimated the effect of chemotherapy on the overall survival (OS) based on the postmatch cohort. With the whole‐population analysis, no significant difference of OS was found between the patients with and without chemotherapy (Figure 2B), implying the assumption of that the patients in the chemotherapy group did not live long enough to develop a SPM could not be established. Moreover, owing to the efficiency and necessity of chemotherapy for tumors at different stages were different, we further analyzed the relationship between chemotherapy and OS according to different tumor stages. The results showed that chemotherapy was not associated with significant changes of OS either (Figure 2C and D). Generally speaking, chemotherapy could play positive roles in the regional‐advanced/advanced stages of HNSCC, such as function preservation,^36^ locoregional tumor control^37^ and extension of survival.^38^ But it is inconsistent in some cases. For example, Amini et.al pointed out comparing with the radiotherapy alone, the concurrent chemoradiotherapy was not associated with longer OS in the subgroups that >81‐year‐old patients as well as 71‐81‐year‐old patients with “T1‐2, N1, and Charlson‐Deyo 0‐1 (CD0‐1) disease” or with “T3‐4, N1+, and CD1+disease.”^39^ Giacalone et.al found that no significant difference of 3‐year OS was observed between patients receiving adjuvant chemoradiotherapy and receiving adjuvant radiotherapy alone in 1686 elderly patients.^40^

There are several limitations in our study: First, this study is retrospective and a selective bias^11^ might be inevitable in a postmatch analysis cohort created by PSM. Second, some important variables such as detailed antitumor regimens, duration of chemotherapy, treatment‐related mortality (especially in older population ^41^) as well as other important risk factors such as smoking, heavy drinking and HPV infection ^42^ were not existed in the SEER database. Third, a clear relationship between SPMs and OS is still ambiguous (Figure S3), so that well‐designed studies might be needed to clarify this. Fourth, some of the sHRs reported in our article are close to 1, thus the attitude toward the results should be careful. Finally, despite the SEER database covers approximately 30% of the population in the United States,^43^ the findings would be more reliable if external validations from other independent large‐scale database were performed.

## 5 CONCLUSIONS

Based on the postmatch cohort after PSM, we found SCC, increased age, male, and well/moderate histological grade were identified as independent risk factors and that chemotherapy was an independent protective factor for SPMs in patients with HNC. Furthermore, a reliable nomogram on the prematch cohort was established not only predicted the 3‐, 5‐, and 10‐year probabilities of SPMs, but also indicated the high‐risk patients characterized as SCC, increased age, male, and well/moderate histological grade were more likely to be benefitted from chemotherapy to avoid SPMs. Unfortunately, no significant difference of OS was observed between the patients with and without chemotherapy. However, further validation by well‐designed trials is needed to generalize the applicability of our results in clinical practice.

## CONFLICT OF INTEREST DISCLOSURES

The authors made no disclosures.

## AUTHORS’ CONTRIBUTIONS

Xinrong Li was involved in conceptualization, formal analysis, interpretation, writing—original draft, and writing—critical revision. Yong Guo was involved in conceptualization, methodology, writing—critical revision, resource provision, and supervision. Kaibo Guo was involved in methodology, data analysis, and interpretation. Yuqian Feng was involved in figures and tables editing.

## Supporting information

Fig S1

Fig S2

Fig S3

## Data Availability

The data that support the findings of this study are available from the corresponding author upon reasonable request.
