# Supplementary figures and images for: Analysis of chemotherapy effect on the second primary malignancy for head and neck cancer patients by a nomogram based on SEER database
Source: Cancer Med. 2020 Sep 15;9(21):8029–42. doi: 10.1002/cam4.3442 (PMC7643691; doi:10.1002/cam4.3442)

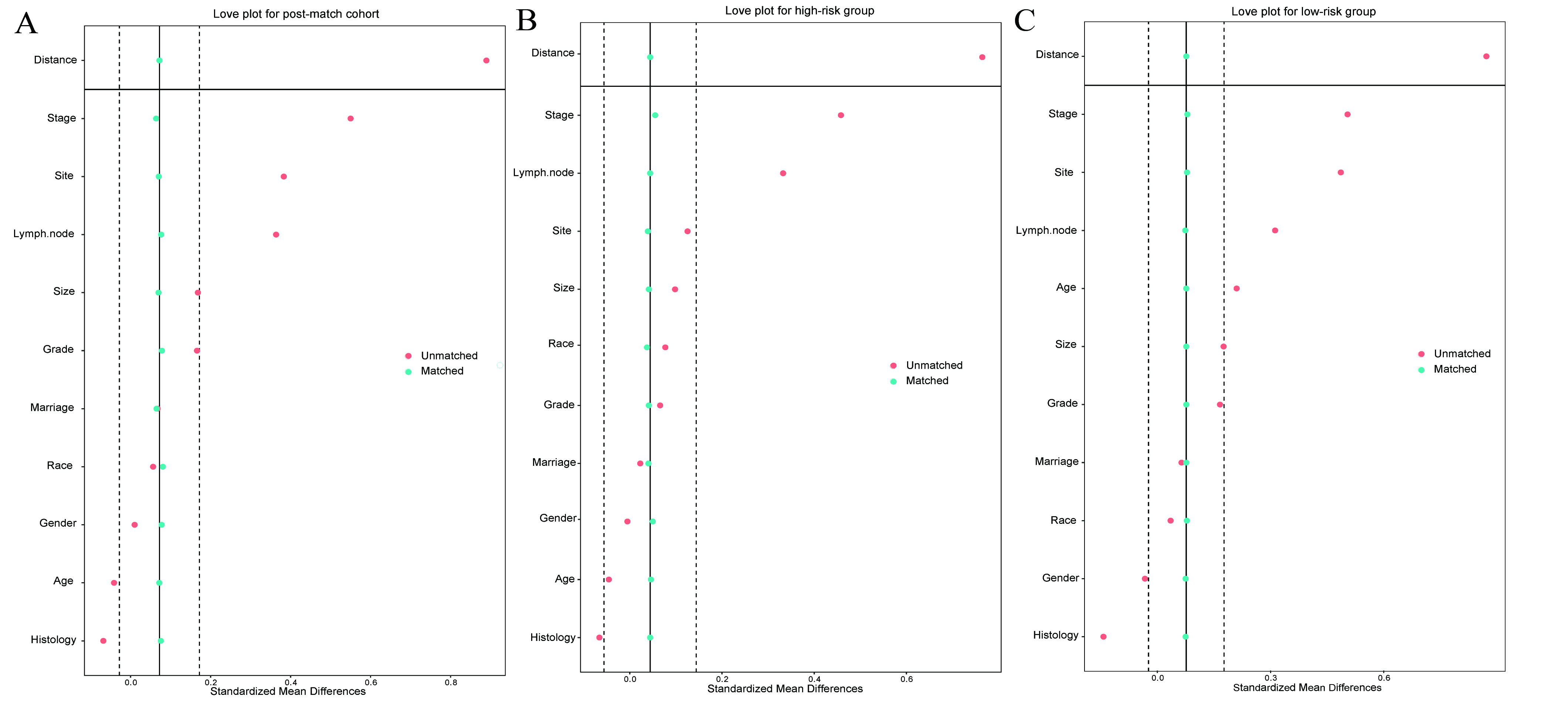

Supplement: Supplementary file 1 — Fig S1 [file CAM4-9-8029-s001.tif]

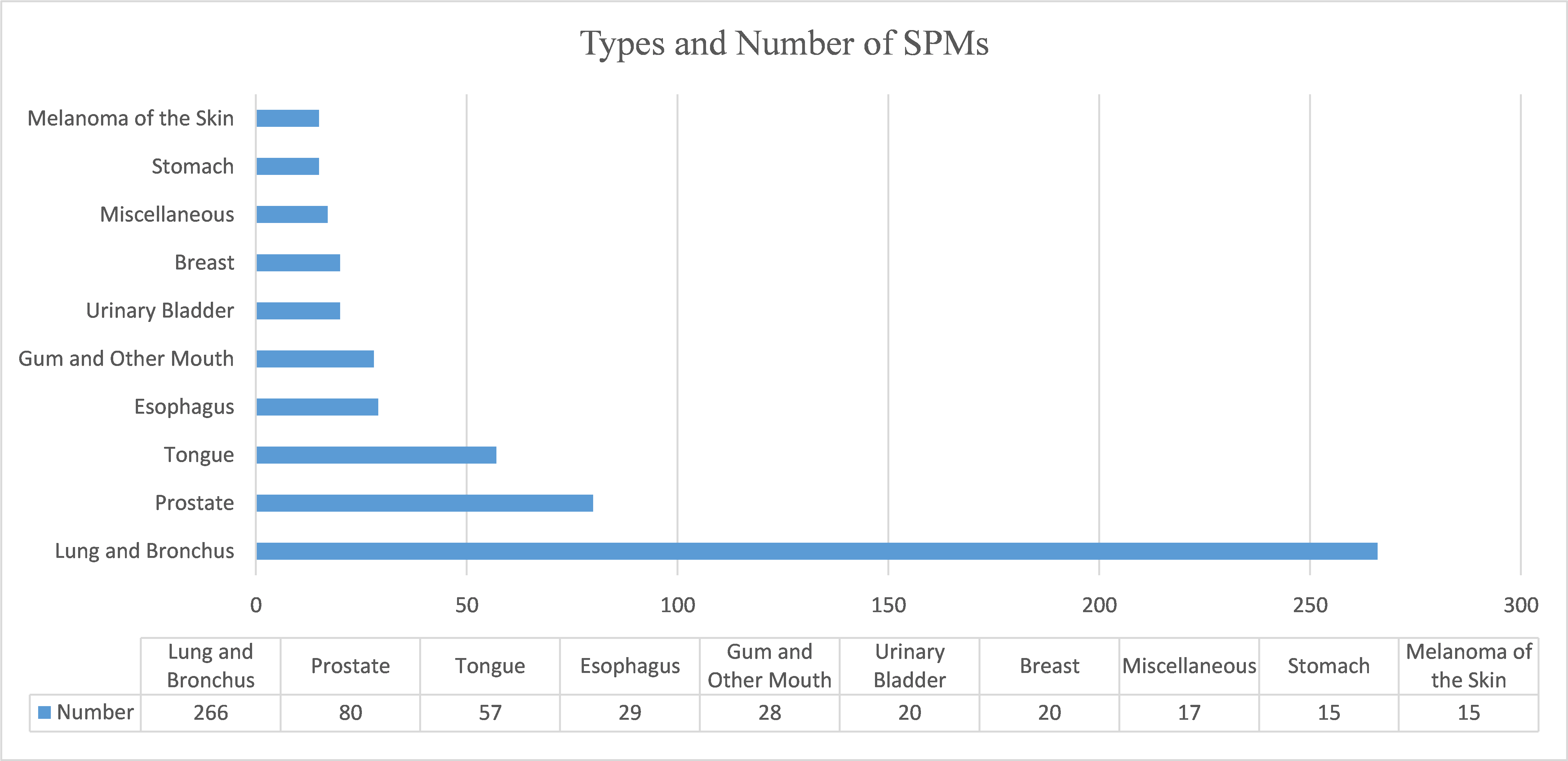

Supplement: Supplementary file 2 — Fig S2 [file CAM4-9-8029-s002.tif]

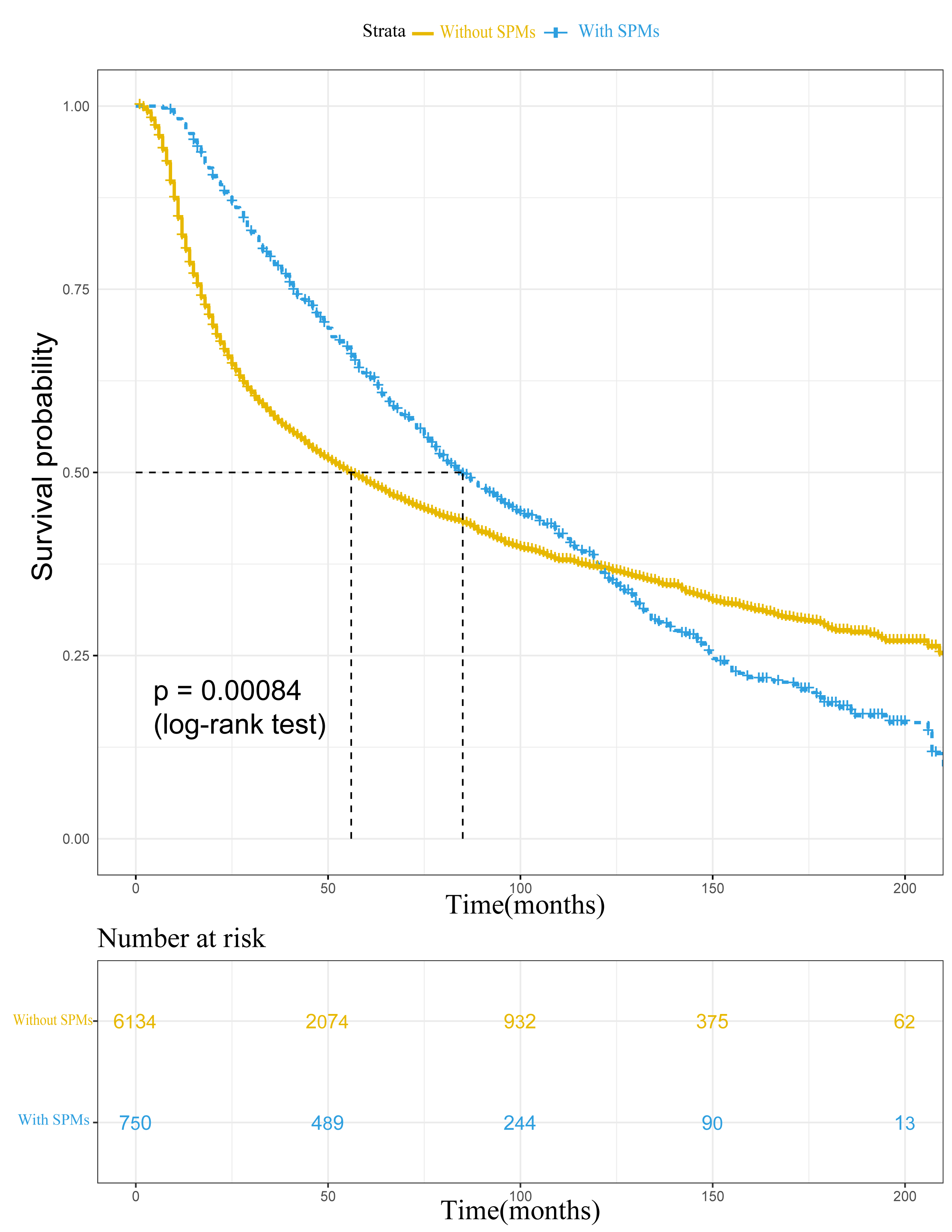

Supplement: Supplementary file 3 — Fig S3 [file CAM4-9-8029-s003.tif]
